# Granulation of m^1^A-modified mRNAs protects their functionality through cellular stress

**DOI:** 10.1093/jmcb/mjaa073

**Published:** 2020-12-30

**Authors:** Hao Jiang

**Affiliations:** Department of Biochemistry and Molecular Genetics, University of Virginia School of Medicine, Charlottesville, VA 22908, USA

To cope with various stress conditions, cells turn off house-keeping processes and turn on stress-protective pathways (Rabouille and Alberti, 2017). A successful strategy would also require cells to efficiently reactivate the essential cellular processes once the stress is over. In addition to activation of signaling events, recent studies have shown that formation of certain membraneless assemblies plays a role in stress adaptation (Rabouille and Alberti, 2017). One prominent type of these assemblies is stress granules (SGs), which are composed of translation-arrested messenger RNAs (mRNAs) and many RNA-binding proteins. The assembly of SGs and other ribonucleoprotein granules is driven by numerous interactions of RNA‒protein, protein‒protein, and RNA‒RNA molecules (Protter and Parker, 2016). Liquid‒liquid phase separation is now considered a key mechanism underlying the dynamic and reversible features of these assemblies, which may facilitate the rapid recovery of the molecular activities after stress (Franzmann et al., 2018).

The diverse chemical modifications of RNAs greatly expand the functional impact and regulatory roles of RNAs in the flow of genetic information. These modifications can also affect the fate of the RNA molecules in response to stress. N6-adenine methylation (m^6^A) is the most abundant mRNA modification, and polymethylated mRNAs can act as a multivalent scaffold for m^6^A-binding proteins, thereby enhancing phase separation of these proteins (Ries et al., 2019). Both the m^6^A mRNAs and their binding proteins are enriched in SGs and functionally promote the formation of SGs (Fu and Zhuang, 2020).

N1-methyladenosine (m^1^A), a modification known earlier in ribosomal RNAs and transfer RNAs, was more recently shown to be also present on mammalian mRNAs (Xiong et al., 2018). A specific sequence motif forming a T-loop mimic in mRNAs is recognized by the TRMT6/61A (or TRMT61B/10C in mitochondria) enzyme complex and subsequently methylated at the N1 position of the adenine base (Xiong et al., 2018). While m^1^A is dynamically regulated in response to developmental and environmental changes, the functional role of m^1^A on mRNAs is poorly understood.

**Figure 1:**
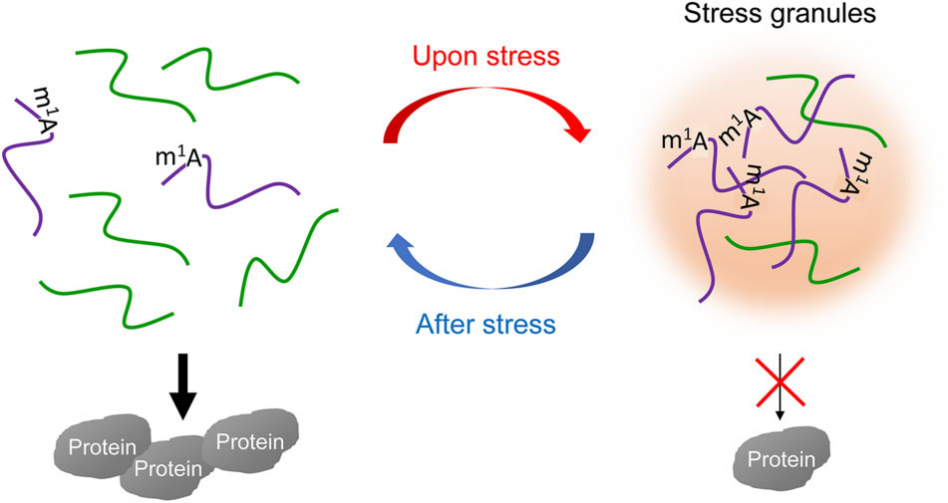
m^1^A-mRNAs are relatively enriched in SGs upon stress but can rapidly return to the translatable pool after stress.

Following their previous finding that TRMT6 and TRMT61A are greatly enriched on free mRNA in heat-shocked cell lysates (Alriquet et al., 2019), Alriquet et al. (2020) showed that mRNAs containing the m^1^A motif were significantly enriched in SGs (Figure 1). They also experimentally demonstrated that both TRMT6/61A and m^1^A accumulated in SGs upon oxidative stress or heat shock. The authors further provided reasoning that the SG-enriched m^1^A was likely to be mostly on mRNAs. It is noteworthy that, while m^6^A was also enriched in SGs upon stress, the SG enrichment of m^1^A was stronger than m^6^A. Depletion of TRMT6/61A reduced m^1^A levels in the cell, impaired stress-induced SG formation, and sensitized cells to oxidative stress or heat shock. These results suggest that TRMT6/61A-catalyzed m^1^A on mRNAs promotes cellular stress response.

How does m^1^A affect the fate of the modified mRNA during stress? To address this challenging question, the authors designed a reporter system, in which they inserted a single m^1^A motif in the 5′ untranslated region of a sequence encoding a highly unstable protein. Meanwhile, they also prepared a mutated reporter containing a thymine instead of adenine in the motif loop to prevent m^1^A modification. They found that the reporter protein level was similar from both reporters under unstressed conditions. This is an important control, as m^1^A may affect mRNA translation (Xiong et al., 2018). They then put the cells through heat shock before returning to normal temperature. While the protein level from the wild-type reporter was higher than that from the mutant immediately after the heat shock, it accumulated significantly faster than that from the unmethylatable mutant reporter during the recovery period after the heat shock (Figure 1). These results suggest that the m^1^A-mRNA responds to heat shock more efficiently in both ways, including the efficient shutdown of its translational activity upon heat and also the rapid returning to normal translation after the heat stress is over.

The data in this study collectively suggest that m^1^A modification on mRNAs promotes an efficient response to cellular stress by partitioning into SGs, which appear to provide dynamic compartments not only for turning off protein synthesis from these transcripts but also for a rapid recovery to enhance the cellular fitness through stressful conditions. This study also opens up many interesting questions, including how m^1^A facilitates RNA sequestration in SGs and how the activities of the endogenous m^1^A-mRNAs respond to stress and recover after stress.
